# A human specific Alu DNA cassette is found flanking the genes of transcription factor AP2

**DOI:** 10.1186/s13104-019-4247-7

**Published:** 2019-04-11

**Authors:** Hamdi K. Hamdi, Siddana Reddy, Nada Laz, Renad Eltaher, Zahraa Kandell, Teif Mahmud, Lamia Alenazi, Basheer Haroun, Mohanad Hassan, Raju Ragavendra

**Affiliations:** Basic Medical Sciences Dept., College of Dentistry, Almustqbal University, PO Box 156, Buraida, Qassim 51411 Saudi Arabia

**Keywords:** Alu, Polymorphic, TFAP2B, TFAP2D, AP2, Dental, Neural, Breast cancer

## Abstract

**Objective:**

Alu elements are retroposons that invaded the primate genome and shaped its biology. Some Alus inserted recently and are polymorphic in the human population. It is these Alus that are being sought after in disease association studies and regulatory biology. Discovering polymorphic Alus in the human genome can open areas of new research in these fields.

**Results:**

Using the polymerase chain reaction on genomic DNA, we identified a polymorphic Alu in the flanking region of the TFAP2B and TFAP2D genes. The new insert was found in higher frequency in Europeans (0.4) and Asians (0.38) and lower frequency in Africans (0.25). We also show this Alu to be part of a 3 Alu cassette that is human specific. The TFAP2B and TFAP2D genes encode members of the transcription factor AP-2, which plays a role in organ development. The insertion of this Alu cassette flanking the transcription factor genes distinguishes humans from the primates. This cassette can possibly affect the regulation of both genes or alternately provoke genomic deletions, which we have shown in this study. Its presence in such a location is intriguing and unquestionably opens an investigational window in disease association studies and in the field of gene regulation.

**Electronic supplementary material:**

The online version of this article (10.1186/s13104-019-4247-7) contains supplementary material, which is available to authorized users.

## Introduction

The Transcription Factor AP2 Beta and Delta (TFAP2B and TFAP2D) genes are located on chromosome 6 and encode members of the AP-2 protein family [1, 2]. AP-2 is a transcription factor that binds directly to DNA either as a homo or hetero dimer regulating gene expression [3]. They display differential binding affinities for different gene promoters and function in both inducing and repressing transcription [4–6]. The AP-2 protein has been shown to be expressed in various tissues including adrenal, esophagus, kidney, placenta, salivary gland, skin and testis [7]. Mutations in these genes have also been discovered in patients with Char syndrome (TFAP2B) [8, 9]. More recently it was shown that mutations in TFAP2B are linked with isolated tooth agenesis, microdontia, supernumerary tooth and tooth root maldevelopment [10]. It has also been shown that the TFAP2B gene product is tumor promoting in breast cancer and appears to be highly expressed in breast cancer cell lines and in tumor tissues of breast cancer patients [11]. Lobular carcinoma in situ and invasive lobular breast cancer also demonstrated enhanced expression of transcription factor AP-2 [12]. In this paper, we present a polymorphic Alu (Arthrobacter luteus restriction enzyme characterized) element insert found flanking both the TFAP2B and TFAP2D genes in humans.

Alu inserts are DNA elements that have invaded the primate genome by retroposition and are now permanent residents in it with over a million interspersed in the human genome [13, 14]. They have contributed to disease by inserting in coding regions, upsetting gene structure [15, 16]. Since coding sequences are less abundant, Alus litter the landscape of non-coding DNA. This contributes to their recruitment for regulatory functions [17, 18] as in the case of the angiotensin converting enzyme (ACE) Alu [19]. Depending on when these Alus have inserted, they can be traced in lineage by their shared descent in the primates at various loci [20]. Alus that are shared between the primate species are used in the phylogenetic studies of primates [20]. Some Alus are of a more recent origin and can be found to be polymorphic in the human population [14, 21, 22]. It is these Alus that are sought after in disease association studies. Thus, the aim of this study is to identify polymorphic Alu elements in the human population, which can be subsequently used in disease association studies. In this paper, we identified a polymorphic Alu element (HK1) flanking the TFAP gene family.

## Main text

### Methods

#### Genomic DNA

Genomic DNA used in this study was obtained from human primary cell cultures, which have been previously described [19]. The primary cultures of human endothelial cells came from a variety of tissues, which were purchased from the American Type Culture Collection (ATCC). The cells came from the following tissues of origin: umbilical vein; pulmonary vein; iliac vein; abdominal aorta; femoral artery; iliac artery; and pulmonary artery.

#### Polymerase chain reaction (PCR)

Total genomic DNA (0.3 µg) was amplified by polymerase chain reaction (PCR) in a 30 µl volume, which included: 20 mM Tris–HCl (pH 8.3), 50 mM KCl, 2.5 mM MgCl2, 250 µM dNTP, 0.2 µM each of forward (5′-GCAGTCCAAACTCGGAAGACTGGG-3′) or reverse (5′-GGACAGAAAGAG GCCTGGACTAC-3′) and 5% dimethylsulfoxide (DMSO). Amplification of DNA was performed at 96 °C for 2 min, followed by 35 cycles of denaturation (96 °C for 30 s), annealing (62 °C for 30 s), and elongation (68 °C for 30 s). The last cycle was followed by a final elongation step (65 °C for 5 min). The reactions were stopped by the addition of 3 µl of a 0.25 M EDTA solution at 0 °C, and aliquots were electrophoresed on a 2% (w/v) agarose gel with 0.5 µg/ml ethidium bromide. The resulting bands were scored for the presence of the Alu and sequenced to confirm their identity. The native (empty site) allele was identified by the presence of a single 271 bp PCR product. The insertion allele (Alu) was identified by the presence of a single 582 bp PCR product.

#### Frequency analysis of the HK1 polymorphic Alu in Genbank’s genomic assemblies

To determine the Allele frequencies for this locus, we screened Genbank’s human genomic database. The Basic local alignment search tool (BLAST) was used to screen the HK1 locus in both complete and partial genomic assemblies [23]. Aligned sequences were included in the data set and filtered to remove redundant alignments from assemblies derived from the same BioSample. Using the BLAST tool as a virtual polymerase chain reaction (vPCR) we screened Genbank assemblies for the HK1 locus.

### Results

**Fig. 1:**
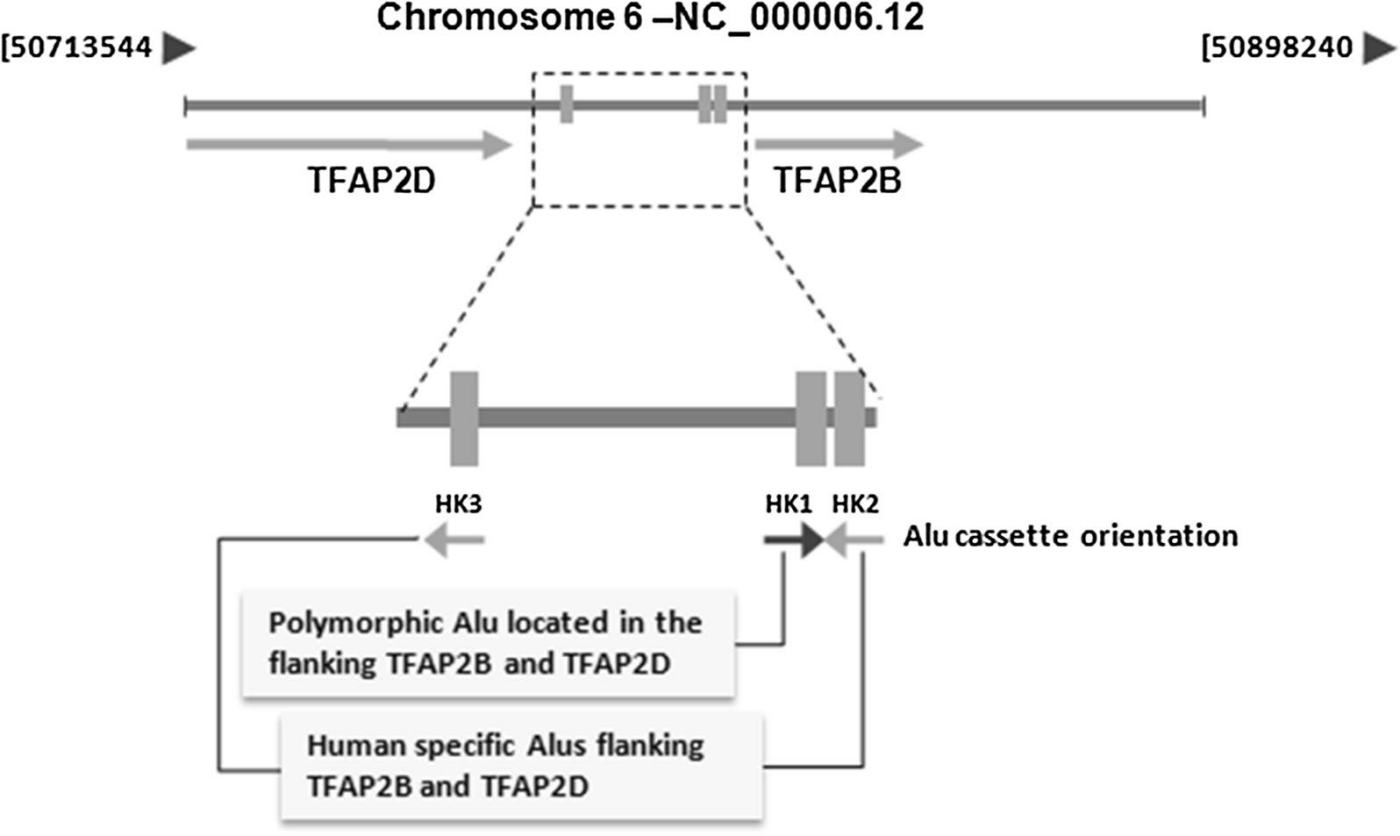
Mapping of the polymorphic HK1 Alu. The polymorphic Alu (HK1) is located in the flanking region of TFAP2B and TFAP2D genes. The HK1 Alu is flanked by 2 other Alu elements (HK2 and HK3) that are human specific and in opposite orientation to HK1

**Fig. 2:**
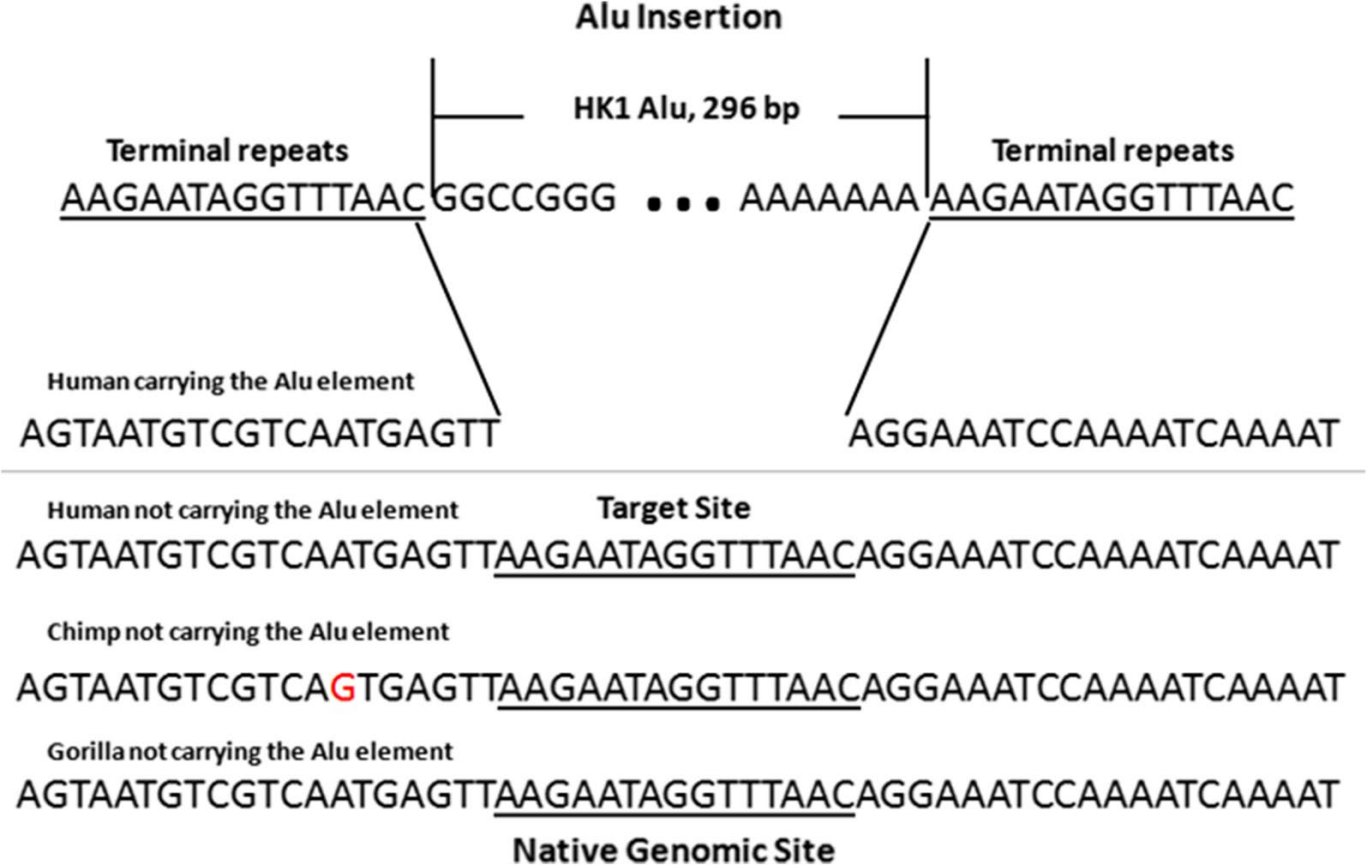
Sequence analysis of the HK1 polymorphic Alu insertion. Shown are aligned sequences from the region surrounding the unoccupied (native genomic site) in humans, Chimp and Gorilla and occupied (HK1 Alu) Alu insertion site in humans. Terminal repeats and target site are underlined

Using Genbank’s map viewer we were able to localize the polymorphic Alu (HK1) in the flanking region of the TFAP2B and TFAP2D genes (Fig. 1). Our PCR amplifications and sequencing yielded 2 alleles for this genomic site. These included the insertion allele and that of the native site. Most Alu elements are retroposons and thus have the hallmark of their RNA origin. Newly inserted Alus have a long poly A tail and are flanked by direct repeats on each end, which is a feature of their insertion at a staggered cut in the DNA. The Alu element we identified was flanked by a poly A tail and direct repeats (Fig. 2). The native site was found in all non-human primates (Fig. 2). The sequences for both alleles were deposited with GenBank under accession numbers: MK574933 and MK574934.

**Fig. 3:**
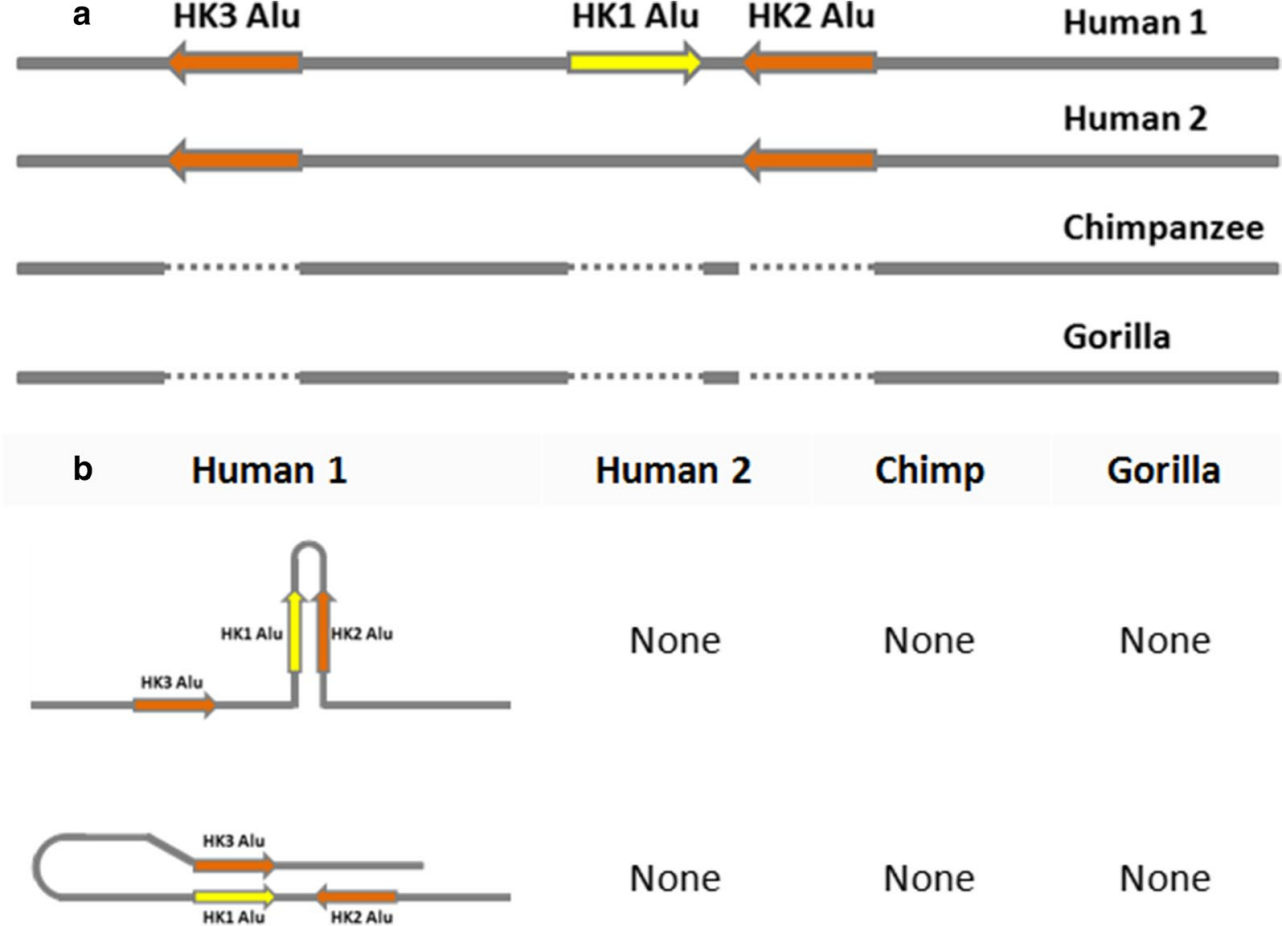
Alu DNA status and orientation flanking the TFAP genes in the primates. All 3 Alu elements are specific to the human and are not present in chimps or gorillas (**a**). The polymorphic HK1 Alu is opposite in orientation to the other 2. All 3 Alus are highly homologous with 96% identity and are potentially capable of secondary structure formation, examples shown in (**b**)

While the goal of this study was not to assess the frequency of the insert in human populations, we conducted a preliminary screen of the polymorphic Alu in Genbank genomic assemblies. It was found in a higher frequency in European (n = 10, 0.40) and Asian populations (n = 16, 0.38) and at a much lower frequency in Africans (n = 16, 0.25). The complete data set is presented as Additional file 1. These numbers, however, should be taken with care due to the limitation in sample size. Although the functional role of this Alu remains to be elucidated, we have found it to be flanked by other Alu elements in reverse orientation forming a cassette of Alus (Fig. 3). Both of the flanking Alus (HK2 and HK3) are human specific and are not found in the primates (Genbank genomic database). The 3 Alus in this cassette are highly homologous, displaying 96% identity and have the potential for intrastrand complementation and secondary structure formation (Fig. 3). Perhaps this configuration can lead to enhancer activity affecting the regulation of TFAP2B and TFAP2D or alternately can play a role in provoking genomic deletions. In fact, we detected such deletions in 2 of Genbank’s genomic assemblies (GCA_000005465.1 and GCA_003391555.1). The deletion in GCA_000005465.1 was bracketed and involved the HK1 and HK2 Alus. While in GCA_003391555.1 a partial deletion of the HK1 Alu was observed.

### Discussion

Alu invasions occurred en masse in the distant past. More recently, however, there is a steady but perhaps slower spread in the collective primate genome [21]. Alus have been studied ever since the advent of genomics, because in fact they make up a significant chunk of the genome. In the beginning, unfortunately, they were labeled as junk DNA to distinguish them from the genic functional parts of the DNA and this has driven their use simply as time markers for studying primate evolution [14, 20]. Over the past decade, however, Alu elements have been recognized as genomic engineers in their own right and their study shifted to the realm of biological function [17, 18, 24, 25]. It has also become obvious that evolutionary change is not limited to the 2% difference in sequence homology between the higher primates but is in fact driven by micro and macro engineering of genomic structure [26]. To distinguish the rapidity of human change from the rest of the primates, it is pertinent to identify other mechanisms of evolutionary change besides that of the single nucleotide polymorphism (SNP) [20]. Here we show a 3 Alu cassette present in the flanking region of TFAP2B and TFAP2D genes. These Alus are all human specific with one being polymorphic in the human population and in opposite orientation to the others. They display high homology to one another having the potential to change local genomic configuration by the formation of DNA secondary structure. The potential for forming this genomic configuration, however, is not available to all humans, since the polymorphic HK1 Alu is a requisite for this formation. Despite the fact that humans carry the HK2 and HK3 Alus, the genomic configuration is not formed unless the cassette is completed with the HK1 Alu, which only some humans carry. In some ways, we can view this genetic system at 3 hierarchical levels. The first level is represented by the primates, which lack all together this cassette and have no potential in accessing this particular genomic configuration. The second level is represented by those humans that carry the HK2 and HK3 Alus, and we can consider them evolutionarily “primed” for this genomic configuration. Finally, humans that carry the full 3 Alu cassette can be considered evolutionarily in an “active state” fully accessing this genomic configuration with all of its positive and negative outcomes. It is in this category that we have discovered genomic deletion events. This discovery sheds light on the continuing dynamic changes occurring in the human genome. It has been shown previously that Alu elements can influence gene expression by either binding transcription factors, forming chromatin looping or secondary structures and even behaving as enhancer elements inducing or repressing transcription [18, 27]. Since they are widely dispersed in the genome, they have the ability to exert powerful regulation on a local as well as a global level. Perhaps the saltatory argument of evolution comes within reach by considering the changes that might occur in the regulation of genes that code for transcription factors. Since transcription factors have a global effect on gene regulation by impacting the expression of hundreds of genes, a small genomic change in their regulation can have an amplifying effect on biology. The insertion of this Alu cassette flanking the transcription factor genes produces a change in the human genome distinguishing humans from primates. If this genomic change leads to a specific human change in the regulation of the AP2 transcription factor (regulating the regulator), this can have an amplifying effect on many genes. The flip side of the argument would be the deleterious effect of Alu elements. Perhaps the presence of the Alu cassette we described might lead to genomic rearrangements/deletions as a result of Alu recombination events resulting in disease outcomes. In this paper, we have seen such deletions; their impact on human biology, however, remains to be elucidated. Regardless of the implied biological role for this Alu cassette, this discovery opens research opportunities in various fields including gene regulation as well as disease association studies.

### Conclusion

By identifying strategically located polymorphic Alu sequences in the human genome, we hope to open a doorway to better understand the etiology of human disease and perhaps the driving force of human evolution. After all, one of the most studied genetic polymorphisms in the history of genetic association studies is the ACE ID polymorphism, which is a polymorphic Alu element present in the angiotensin converting enzyme gene [28]. This Alu polymorphism alone has led to over 3700 publications (as of February 2019) and has been shown to associate with varied diseases in the human population [28]. Here we present a newly inserted Alu element flanking the human TFAP2B and TFAP2D genes and like the ACE Alu is also polymorphic in the human population. More interestingly, however, the HK1 Alu is part of a 3 Alu human specific cassette with the potential of reconfiguring chromatin structure.

## Limitations

Although we have found a polymorphic Alu element flanking the genes TFAP2B and 2D, the allelic frequencies were based on a small set of alleles and should be treated with caution. A larger population study of this polymorphic Alu should be conducted across a wide section of the human population to better approximate the allelic frequencies.

## Additional file

**Additional file 1: Table S1.** Distribution of HK1 polymorphic Alu in GenBank Genomic Assemblies.

## Abbreviations

ACE ID: angiotensin converting enzyme insertion-deletion polymorphism
TFAP2B: transcription factor AP2 beta gene
TFAP2D: transcription factor AP2 delta gene
ATCC: American Type Culture Collection
