# Supplementary material for: A human specific Alu DNA cassette is found flanking the genes of transcription factor AP2
Source: BMC Res Notes. 2019 Apr 11;12:222. doi: 10.1186/s13104-019-4247-7 (PMC6458609; doi:10.1186/s13104-019-4247-7)
Supplement: Supplementary file 1 — Additional file 1: Table S1. Distribution of HK1 polymorphic Alu in GenBank Genomic Assemblies. [file 13104_2019_4247_MOESM1_ESM.docx]

Table S1. Frequency of HK1 polymorphic Alu in GenBank Genomic Assemblies

|  | Assembly | Biosample | Sex | Ethnicity | HK1 Alu status | | Alu+ | Alu¯ |
| --- | --- | --- | --- | --- | --- | --- | --- | --- |
| 3 | GCA_000252825.1 | SAMN02981236 | Male | European | Alu^+^/Alu¯ | | 1 | 1 |
| 4 | GCA_000306695.2 | SAMN02205338 | NA | NA | Alu ¯ | |  | 1 |
| 5 | GCA_000365445.1 | SAMN02981219 | Male | NA | Alu^+^ | | 1 |  |
| 6 | GCA_001292825.2 | SAMN00009513 | Male | NA | Alu ¯ | |  | 1 |
| 7 | GCA_001524155.4 | SAMN03838746 | Female | African | Alu^+^ | | 1 |  |
| 8 | GCA_001712695.1 | SAMN04018081 | Male | Korean | Alu^+^ | | 1 |  |
| 9 | GCA_002077035.3 | SAMN05181962 | Female | Mormon | Alu ¯ | |  | 1 |
| 10 | GCA_002180035.3 | SAMN04229552 | Female | Han Chinese | Alu ¯ | |  | 1 |
| 11 | GCA_003634875.1 | SAMN10182268 | Female | Puerto Rican | Alu ¯/ Alu ¯ | |  | 2 |
| 12 | GCA_000004845.2 | SAMN00003318 | Male | Chinese | Alu ¯ | |  | 1 |
| 13 | GCA_000005465.1 | NA18507 | Male | African | Alu^+^/ Deletion | |  |  |
| 14 | GCA_000185165.1 | SAMN02981291 | Female | NA | Alu ¯ | |  | 1 |
| 15 | GCA_001013985.1 | SAMN02902633 | Female | NA | Alu ¯ | |  | 1 |
| 19 | GCA_001483585.1 | SAMN04316313 | Female | NA | Alu ¯ | |  | 1 |
| 20 | GCA_001708065.2 | SAMN04251426 | Male | Chinese | Alu ¯ | |  | 1 |
| 21 | GCA_002022525.1 | SAMN05589122 | Male | Chinese | Alu ¯/ Alu ¯ | |  | 2 |
| 22 | GCA_002022805.1 | SAMN05589119 | Female | African | Alu ¯/ Alu ¯ | |  | 2 |
| 23 | GCA_002022825.1 | SAMN05589124 | Male | European | Alu^+^/Alu¯ | | 1 | 1 |
| 24 | GCA_002022845.1 | SAMN03274119 | Female | NA | Alu ¯/ Alu ¯ | |  | 2 |
| 25 | GCA_002022865.1 | SAMN05589121 | Female | Puerto Rican | Alu ¯/ Alu ¯ | |  | 2 |
| 26 | GCA_002022975.1 | SAMN05589120 | Female | African | Alu^+^/ Alu^+^ | | 2 |  |
| 27 | GCA_002023025.1 | SAMN05589123 | Male | Askenazi | Alu ¯/ Alu ¯ | |  | 2 |
| 41 | GCA_003382745.1 | SAMN00000528 | Male | Japanese | Alu^+^/ Alu^+^ | | 2 |  |
| 42 | GCA_003384065.1 | SAMN00001168 | Female | African | Alu ¯/ Alu ¯ | |  | 2 |
| 43 | GCA_003384745.1 | SAMN00007798 | Male | Mexican | Alu ¯/ Alu ¯ | |  | 2 |
| 44 | GCA_003391435.1 | SAMN00007969 | Female | Indian | Alu ¯/ Alu ¯ | |  | 2 |
| 45 | GCA_003391465.1 | SAMN00007822 | Female | African | Alu ¯/ Alu ¯ | |  | 2 |
| 52 | GCA_003391495.1 | SAMN00009091 | Female | European | Alu^+^/ Alu^+^ | | 2 |  |
| 53 | GCA_003391525.1 | SAMN00006405 | Female | European | Alu ¯/ Alu ¯ | |  | 2 |
| 54 | GCA_003391555.1 | SAMN00249889 | Female | Chinese | Alu^+^/ Alu^+^  deletetion | | 2 |  |
| 55 | GCA_003391745.1 | SAMN00779963 | Male | African | Alu ¯/ Alu ¯ |  | | 2 |
| 56 | GCA_003391775.1 | SAMN00249724 | Female | Peruvian | Alu^+^/ Alu^+^ | 2 | |  |
| 57 | GCA_003391825.1 | SAMN01036809 | Male | African | Alu ¯/ Alu ¯ |  | | 2 |
| 58 | GCA_003391855.1 | SAMN01090994 | Female | Tamil | Alu ¯/ Alu ¯ |  | | 2 |
| 59 | GCA_003391885.1 | SAMN00001600 | Female | Chinese | Alu ¯/ Alu ¯ |  | | 2 |
| 60 | GCA_003391915.1 | SAMN00001268 | Female | European | Alu ¯/ Alu ¯ |  | | 2 |
| 63 | GCA_001297185.2 | SAMN02744161 | NA | NA | Alu ¯ |  | | 1 |
| 64 | GCA_001542345.1 | SAMN03283347 | Male | Ashkenazi | Alu ¯ |  | | 1 |
| 65 | GCA_001856745.1 | SAMN05213946 | Male | Chinese | Alu ¯ |  | | 1 |
| 66 | GCA_002009925.1 | SAMN04169050 | Male | Asian | Alu^+^/Alu¯ | 1 | | 1 |
| 67 | GCA_002208065.1 | SAMN04229548 | Female | Puerto Rican | Alu ¯ |  | | 1 |
| 68 | GCA_002209525.2 | SAMN05603729 | Female | Colombian | Alu^+^ | 1 | |  |
| 69 | GCA_002872155.1 | SAMN06885952 | Female | African | Alu ¯ |  | | 1 |
| 71 | GCA_002884485.1 | SAMN03255769 | Female | NA | Alu ¯ |  | | 1 |
| 72 | GCA_003070785.1 | SAMN05603847 | Female | Vietnamese | Alu ¯ |  | | 1 |
| 73 | GCA_003086635.1 | SAMN08723473 | Female | African | Alu ¯ |  | | 1 |
| 74 | GCA_003574075.1 | SAMN05603745 | Female | African | Alu^+^ | 1 | |  |
| 75 | GCA_003601015.1 | SAMN10026989 | Female | Bengali | Alu^+^ | 1 | |  |
| 76 | GCA_900232925.2 | SAMEA104349931 | NA | NA | Alu ¯ |  | | 1 |
